# The Spider Venom Peptide Lycosin-II Has Potent Antimicrobial Activity against Clinically Isolated Bacteria

**DOI:** 10.3390/toxins8050119

**Published:** 2016-04-26

**Authors:** Yongjun Wang, Ling Wang, Huali Yang, Haoliang Xiao, Athar Farooq, Zhonghua Liu, Min Hu, Xiaoliu Shi

**Affiliations:** 1 Department of Medical Genetics, 2nd XiangYa Hospital of Central South University, Changsha 410011, China; tsm2076@yeah.net (Y.W.); tracytsm2009@163.com (H.Y.); 2 Department of Gastroenterology, 2nd XiangYa Hospital of Central South University, Changsha 410011, China; 3 Clinical Laboratory, 2nd XiangYa Hospital of Central South University, Changsha 410011, China; wl13787024873@163.com; 4 Medical Experimental Animal Center, 2nd XiangYa Hospital of Central South University, Changsha 410011, China; leon228890575@163.com; 5 College of Life Sciences, Hunan Normal University, Changsha 410081, Hunan, China; drathar.cn@hotmail.com (A.F.); liuzh@hunnu.edu.cn (Z.L.)

**Keywords:** antimicrobial peptides, spider venom, drug-resistant bacteria, cationic α-helical conformation

## Abstract

Antimicrobial peptides have been accepted as excellent candidates for developing novel antibiotics against drug-resistant bacteria. Recent studies indicate that spider venoms are the source for the identification of novel antimicrobial peptides. In the present study, we isolated and characterized an antibacterial peptide named lycosin-II from the venom of the spider *Lycosa singoriensis*. It contains 21 amino acid residue lacking cysteine residues and forms a typical linear amphipathic and cationic α-helical conformation. Lycosin-II displays potent bacteriostatic effect on the tested drug-resistant bacterial strains isolated from hospital patients, including multidrug-resistant *A. baumannii*, which has presented a huge challenge for the infection therapy. The inhibitory ability of lycosin-II might derive from its binding to cell membrane, because Mg^2+^ could compete with the binding sites to reduce the bacteriostatic potency of lycosin-II. Our data suggest that lycosin-II might be a lead in the development of novel antibiotics for curing drug-resistant bacterial infections.

## 1. Introduction

The antibacterial resistance for many antibiotics used in clinical settings has become one of the most serious public health threats in the world. The development of novel antibiotics overcoming antimicrobial resistance is therefore urgent and in great demand. Antimicrobial peptides (AMPs) have been accepted as powerful novel weapons against the growing number of pathogenic organisms that are resistant to traditional antibiotics. They demonstrate broad-spectrum antimicrobial activity [1,2]. Currently, more than 1000 AMPs have been identified or predicted from nucleic acid sequences in many species from bacteria to mammals [3,4]. Most AMPs are linear cationic α-helical peptides, which lack cysteine residues and are generally short, consisting of <40 amino acid residues. Many of these peptides are disordered in aqueous solutions, whereas they adopt a α-helical conformation in the presence of secondary structure accelerators (trifluoroethanol or sodium dodecylsulphate) and amphiphilic compounds (phospholipid liposome or lipid A) [5,6]. Most studies indicate that the activity of linear cationic α-helical AMPs against microbes is characterized by their ability to disrupt the cell membrane. This mechanism is rather different from that of traditional antibiotics. The bactericidal action of these AMPs is known to be mediated by a stepwise and rapid process. The cell membrane disruption and rapid killing of AMPs make it difficult to develop resistance [5,7,8,9]. Therefore, AMPs have been accepted as good candidates for the development of novel antibiotics.

Spider venoms contain diverse peptide toxins which have attracted great attention as promising drug leads and excellent research tools in pharmacology and neurobiology. Most spider toxins are 30–50 amino acid peptides containing multiple disulfide bonds and function as neurotoxins modulating ionic currents in ion channels, which have been studied extensively [10,11,12]. In recent years, a particular type of spider toxins lacking cysteine residue and with α-helical secondary structure has gained increasing recognition. These interesting peptides are amphipathic and positively charged, and exhibit strong antimicrobial activity via a pore-forming mechanism [13,14,15,16,17,18,19,20]. These peptides belong to the antimicrobial peptide (AMP) family. In our previous studies, we isolated a 24-amino acid peptide named lycosin-I from the venom of the spider *L. singoriensis.* It shows a linear amphipathic α-helical conformation and effective anticancer activity. It also has a rapid, potent and broad-spectrum antimicrobial activity both *in vitro* and *in vivo* [21,22,23]. In the present study, a novel 21 amino acid peptide named lycsoin-II was isolated from the same spider venom. Lycosin-II is also a linear cationic α-helical peptide, and its amino acid sequence is completely different from that of lycosin-I. Interestingly, it displayed potent inhibitory effect on some clinically isolated multi-drug-resistant bacteria.

## 2. Results and Discussion

### 2.1. The Biochemical Properties of Lycosin-II

**Figure 1:**
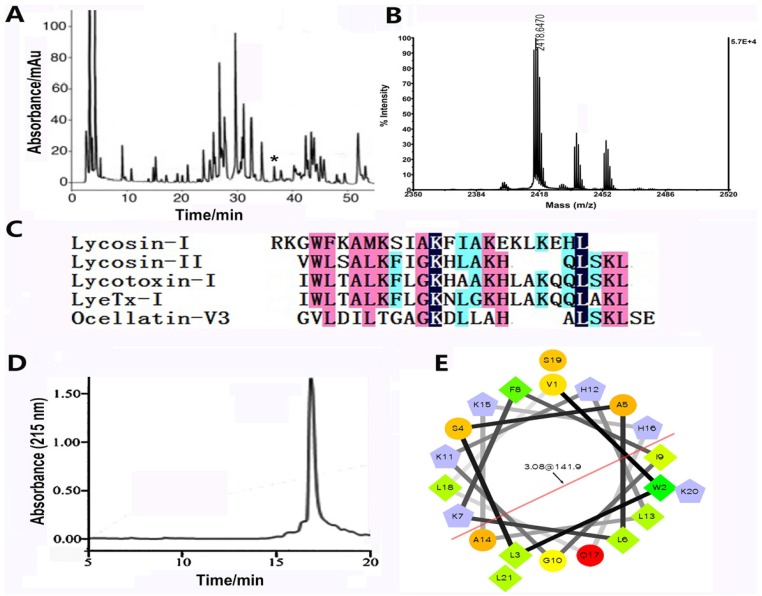
Purification and characterization of lycosin-II. (**A**) Purification of lycosin-II by RP-HPLC (column, Vydac, C18, 300 Å, 4.6 mm× 250 mm). Venom components were eluted using a linear acetonitrile gradient (0%–60% acetonitrile/0.1% TFA in 60 min) at a flow rate of 1.0 ml/min. Elution of peptides was monitored at 215 nm. The peak labeled with an asterisk (*) contains lycosin-II. (**B**) MALDI-TOF MS of lycosin-II. (**C**) Multiple sequence alignment. Lycosin-II shows some similarity with some antimicrobial peptides. (**D**) Purification of synthetic lycosin-II by using RP-HPLC. (**E**) The α-helical wheel projection of lycosin-II, showing the amphipathic and cationic α-helix configuration of lycosin-II.

Our previous study indicated that the crude venom of the wolf spider *L. singoriensis* has antibacterial activity, hinting that the venom might contain antibacterial components [24]. Actually, an antibacterial peptide named lycosin-I was identified in the venom. In order to identify more AMPs from this venom, approximate 10 mg of crude venom was fractionated by using C18 Reverse-Phase High Performance Liquid Chromatography (RP-HPLC). As shown in Figure 1A, the RP-HPLC purification showed that the crude venom is a complex mixture. More than 80 peaks were observed in the chromatography. All the peaks were collected and analyzed by using Matrix-Assisted Laser Desorption/ Ionization Time of Flight Mass Spectrometry (MALDI-TOF MS). The peak labeled with asterisk (*) displayed the average molecular mass as 2418.647 Da (M + H^+^) (Figure 1B). Its amino acid sequence was further determined to be VWLSALKFIGKHLAKHQLSKL, as determined from automatic Edman degradation. As revealed by the cDNA sequence of lycosin-II, residues “GR” were contained at the C-terminal, indicating C-terminal amidation during post-translational process (data not shown). Like lycosin-I, lycosin-II is also a linear peptide without cysteine residues. Another structural feature of lycosin-II is that it contains four lysine residues, which make it a rather basic peptide at physiological pH. In the absence of cysteine residues, lycosin-II does not form inhibitor cystine knot (ICK) motif which is universally adopted by many spider peptide toxins [10]. Although, its amino acid sequence is distinct from that of lycosin-I, lycosin-II showed high sequence similarity with several AMPs from other species (Figure 1C). Lycotoxin-I and LyeTx I are AMPs from the venoms of the wolf spiders *L. carolinensis* and *L. erythrognatha*, respectively [14,20]; Ocellatin-V3 is from the Caribbean frog *Leptodactylus validus* [25]. These three AMPs are also linear cationic α-helical peptides. Similarly, lycosin-II was predicted to adopt α-helix conformation in secondary structure. The α-helical wheel projection of lycosin-II highlighted the most likely configuration of amphipathic and cationic α-helix (Figure 1D). Such structural property indicated that lycosin-II might be antibacterial through acting on cell membrane. Because the level of lycosin-II present in the natural crude venom is extremely low, we prepared the synthetic lycosin-II using Fmoc-solid-phase method, and the synthetic compound has identical molecular mass with that of the native peptide (Figure 1E). The synthetic peptide was used in all the experiments described below.

### 2.2. The Antibacterial Effects of Lycosin-II

**Figure 2:**
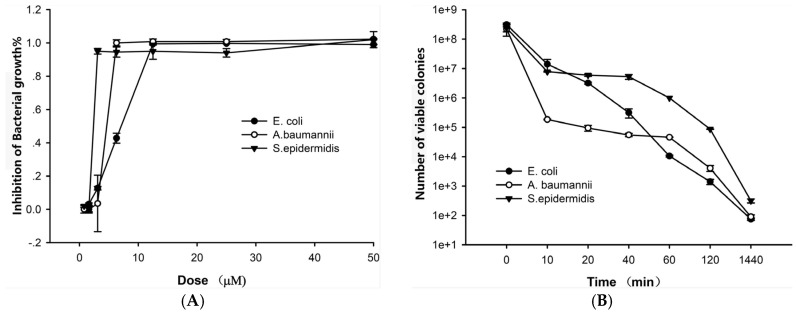
The antibacterial effects of lycosin-II. (**A**) Lycosin-II shows inhibitory effects on three clinical strains *E. coli*, *A. baumannii*, and *S. epidermidis*. The inhibition was dose-dependent. (**B**) Time-dependent bactericidal effect of lycosin-II on the three strains. The numbers of viable colonies were counted at each of the indicated time points. The concentration of lycosin-II used was 50 μM for *E. coli*, 12.5 μM for *A. baumannii*, or 12.5 μM for *S. epidermidis*. All tests were conducted in triplicate. The data were expressed as mean ± SE.

The antibacterial activity of lycosin-II was determined on clinical bacteria strains isolated from ascites or stupa of hospital patients. These bacteria strains were considered as multidrug resistant strains because they were resistant to most conventional clinical antibiotics. They would have potent threats to hospital patients. In fact, most of these patients from whom the bacterial isolates tested were collected were severe patients in Intensive Care Unit (ICU). As shown in Figure 2A, lycosin-II exhibited potent inhibitory effects on the three strains, *Escherichia coli*, *Staphylococcus epidermidis*, and *Acinetobacter baumannii*. The inhibition by the peptide was dose-dependent. At the concentrations of 3.1–12.5 μM, lycosin-II could completely inhibit the growth of these three strains, respectively. The minimum inhibitory concentrations (MICs) of lycosin-II against the bacteria were further determined using a micro dilution assay. A total of 18 strains of bacteria, including 7 Gram-negative bacterial strains from patient ascites, 8 strains of *A. baumannii*, and 3 strains of *Staphylococcus aureus* were tested. The results are shown in Table 1. Lycosin-II was able to inhibit the growth of all bacterial strains with MIC values ranging from 3.1 to 25 μM, depending on the type of bacteria tested. *Staphylococcus saprophyticus*, *S. epidermidis*, *Viridans Streptococci*, *S. aureus*, and *A. baumannii* are the most susceptible to lycosin-II. *Klebsiella pneumoniae* and *Streptococcus pyogenes* were less sensitive to the peptide. Only the highest dose (50 μM) of lycosin-II demonstrated evident inhibitory response.

Exposure to lycosin-II at 4-fold MIC resulted in the immediate death of bacteria, as shown in the time–kill curves (Figure 2B). Three bacterial strains—*E. coli*, *S. epidermidis*, and *A. baumannii*—were examined. The number of visible colonies decreased rapidly during the first 10 min of lycosin-II treatment. Then, a gradual reduction of visible colonies was still observed till 24 h incubation with lycosin-II. These data were consistent with the rapid antibacterial activity of AMPs reported in earlier studies [1,6]. The rapid killing effect on bacteria was probably responsible for the potent growth-inhibitory activity of lycosin-II.

### 2.3. Competition between Lycosin-II and Mg^2+^

**Figure 3:**
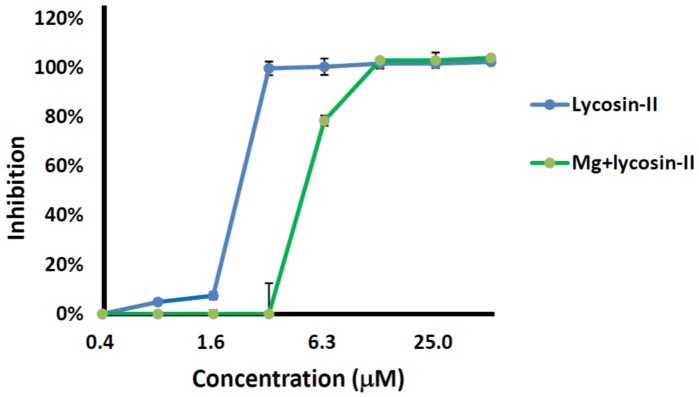
Effect of Mg^2+^ on the antimicrobial activity of lycosin-II against a strain of *A. baumannii.* Bacterial growth was measured at 630 nm. The MIC of lycosin-II was 3.1 μM and 12.5 μM in the absence and presence of 5 mM Mg^2+^, respectively. The assay was performed in triplicate. The data were expressed as mean ± SE.

To clarify the binding of lycosin-II to the bacterial cell membrane, we measured the MIC values of lycosin-II against a strain of *A. baumannii* in the presence or absence of 5 mM Mg^2+^. As shown in Figure 3, Mg^2+^ decreased the inhibitory potency of lycosin-I against the strain. The MIC of lycosin-II was 3.1 μM and 12.5 μM in the absence and presence of 5 mM Mg^2+^, respectively, indicating that Mg^2+^ caused a 4-fold increment in the MIC of lycosin-II. These data suggested that Mg^2+^ might compete for the binding sites on the bacterial surface. This might be explained by which lycosin-II, similar to many other cationic AMPs, could potently bind to the electronegative bacterial cell membrane because of its net positive charge. As is known, the special outer envelopes of Gram-negative bacteria such as *A. baumannii* consist of lipopolysaccharides (LPS), which provide negatively charged binding sites for cationic ions under physiological condition. The abundant Mg^2+^ would rapidly saturate these binding sites and hinder the interaction between the cationic peptide and bacterial cell membranes, especially at low peptide concentrations. On the other hand, although the reduced inhibition of lycosin-II in high concentration of salt was observed, lycosin-II still retained its potency to inhibit the growth of *A. baumannii*, even exposed to 5 mM Mg^2+^, indicating that the antibacterial activity of lycosin-II was moderately salinity-tolerant.

As lycosin-II shares sequence homologies with most cationic antimicrobial peptides, in which the hydrophobic and hydrophilic amino acid residues arrange alternately, it might function as a pore-forming or membrane-permeabilizing peptide, just like these amphipathic α-helical peptides. Three models have been proposed to explain the membrane-permeabilization process—the barrel-stave model, the carpet model and the toroidal-pore model—all of which are based on the mechanisms of action of classic AMPs such as alamethicin, cecropin, melittin, magainins, LL-37, and others [26]. The underlying mechanism of action of lycosin-II likely involves one of the action models. Experiments should be performed to clarify this in the future.

### 2.4. Lycosin-II Has Low Hemolytic Activity

**Figure 4:**
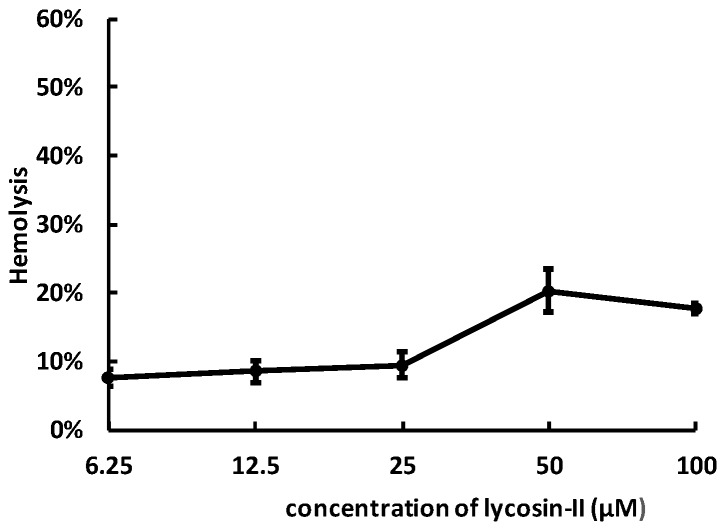
The hemolytic effects of lycosin-II on human erythrocytes. The assay was performed in triplicate. The data were expressed as mean ± SE.

To investigate the toxicity of lycosin-II against mammalian cells, we examined their hemolytic activity on human erythrocytes at different concentrations. The curves in Figure 4 indicate that lycosin-II exerted growing hemolysis with the increase of concentrations. At the concentration of 50 μM, lycosin-II led to approximately 20% hemolysis. Compared with microbe cells, human erythrocytes are less sensitive to lycosin-II.

## 3. Conclusions

In summary, lycosin-II is a native cationic α-helical antibacterial peptide from the venom of the spider *L. singorensis*. It shows potent, broad-spectrum, and rapid antibacterial effect on multi-drug resistant bacterial strains isolated from hospital patients. The bacteriostatic effect of lycosin-II might be correlated with its ability to binding to bacterial cell membrane. The resistance of multidrug-resistant bacteria to most traditional antibiotics results in huge challenges for infection therapy. For example, *A. baumannii* is one of the predominant pathogens associated with nosocomial infections. The misuse of antibiotics has caused the continuous emergence of multidrug-resistant *A. baumannii* isolates. Polymyxins and, possibly, tigecycline are considered to be the last resort of reliable treatments. However, the emergence of multidrug-resistant *A. baumannii* resistance to both antibiotics has been reported worldwide, which has encouraged scientists to develop new alternative agents [27,28,29]. Our data also suggest that lycosin-II would have potential for the development of novel therapeutic agents against infections caused by drug-resistant bacteria, including multidrug-resistant *A. baumannii* isolates. The bacteriostatic effect *in vivo* and the mechanism of action lycosin-II will be determined in our future study. On the other hand, being a linear peptide, lycosin-II might have poor stability due to enzymatic degradation, which makes lycosin-II suffer the short duration of action *in vivo*. This is one of the major obstacles in further application of lycosin-II *in vivo*. Modifications by unnatural amino acid residue replacement or conformational stabilization of the 3D structure might help to improve lycosin-II stability and pharmacokinetics *in vivo*.

## 4. Materials and Methods

### 4.1. Purification, Characterization, and Synthesis of Lycosin-II

The purification, mass determination, amino acid sequence analysis, and synthesis of lycosin-II were performed as previously described. Briefly, the crude venom of the spider *L. singorensis* was fractionated by using RP-HPLC (C18, 4.6 mm × 250 mm, Higgins Analytical, California, CA, USA) via a 0.1% TFA/acetonitrile gradient (0%–60% for 60 min) at 1 mL/min flow rate. The eluted fractions were collected and submitted for mass determination by MALDI-TOF MS (Ultra Flex I, Bruker Daltonics, Karlsruhe, Germany) and for amino acid sequencing by Edman degradation.

For MALDI-TOF-TOF MS analysis, a 1-μL aliquot of each peptide elution was spotted onto a 96-well target plate along with an equal volume of a matrix solution containing 20 mg/mL α-cyano-4-hydroxycinnamic acid (CHCA), 50% ACN, and 0.1% TFA. Mass spectrometry was performed using an acceleration voltage of 25 kV. Monoisotopic molecular mass of each peptide was determined.

Lycosin-II was synthesized using an Fmoc/tert-butyl strategy and HOBt/TBTU/NMM coupling method on an automatic peptide synthesizer (PS3, PTI, Tucson, AZ, USA). Since lycosin-II is *C*-terminally amidated, a poly (ethylene glycol) polystyrene resin equipped with a peptide amide linker Fmoc-peptide amide linker-polyethylene glycol-polystyrene (PAL-PEG-PS) amide resin, Applied Biosystems was used. The Fmoc-amino acids with side chain protection were purchased from GL Biochem Ltd. (Shanghai, China). Peptide synthesis was accomplished on a 0.1-mmol scale. The terminal Fmoc group was removed by treatment with 25% piperidine/*N*,*N*-dimethylformamide (*v/v*).

### 4.2. Bacterial Strains

All clinical isolates tested in this study were collected from the Second Xiangya Hospital during the period between January and July in 2014. *E. coli*, *K. pneumonia*, *S. pyogenes*, *S. saprophyticus*, *S. epidermidis*, *P. aeruginosa*, and *V. streptococci* isolates were collected from patients with ascites. *A. baumannii* and *S. aureus* isolates were collected from patients’ sputum (Medical ethics committee of 2nd XiangYa Hospital of Central South University, S062, 31 December 2013). The identification and antibiotic susceptibility were performed via the MicroscanW/A96 system. These bacterial strains from patient’s ascites were found to be resistant to most clinical conventional antibiotics. *A. baumannii* strains, which were resistant to at least three classes of antibiotics including cephalosporins, carbapenems, β-lactamases, aminoglycosides, and quinolones, were classified as multidrug resistant strains. *S. aureus* strains were resistant to methicillin. For the other strains, drug-resistance was defined as resistance to some of antibiotics including vancomycin, clindamycin, linezolid, teicoplanin, erythromycin, ofloxacin, levofloxacin, oxacillin, and ampicillin. The experiment operation was strictly according to the Clinical and Laboratory Standard Institute (CLIS) criteria.

### 4.3. Minimum Inhibitory Concentration Assay

The minimum inhibitory concentrations (MICs) of lycosin-II were determined by the broth micro-dilution method referring to the CLSI protocol. Bacterial isolates were inoculated into horse blood agar and cultured in 37 °C for 20–24 h to the mid-exponential growth phase. Then, bacteria were diluted with MH broth in 96-well microliter plates to about 5 × 10^5^ CFU/mL (CFU stands for colony-forming units) in a 100-μL final volume supplemented with different concentrations of lycosin-II (50, 25, 12.5, 6.25, 3.1, 1.6, 0.8, and 0 μM/mL). The treatment with 0 μM/mL lycosin-II was considered as the control. The plates were covered with gas-permeable membranes and incubated at 37 °C for 18–20 h. The bacteria growing turbidity in each well was detected at 600 nm (OD_600_). All assays were tested in triplicate; the growth and sterility controls were conducted simultaneously. The percentage of inhibition was calculated according to the following equation: % inhibition = (A_control_ − A_sample_)/A_control_. The MIC was defined as the lowest concentration of lycosin-II causing at least a 90% decrease in turbidity compared to the growth wells.

### 4.4. Time-Kill Kinetics of Lycosin-II

The time-kill curves for lycosin-II were determined against *E. coli*, *A. baumannii*, and *S. epidermidis* strains at 4 × MIC. Briefly, bacteria were grown overnight, diluted in LB broth using flasks with approximately 5 × 10^8^ CFU/mL bacteria, and cultured to the exponential phase. Viable colonies (CFU/mL) were counted at 0, 10, 20, 40, 60, 120, and 1440 min after peptide addition via serial dilution into sterile saline and plating of 0.1 ml of serial dilutions onto LB agar. All agar plates were incubated for 24 h at 37 °C.

### 4.5. Salt Tolerance

To determine the effect of Mg^2+^ on the antibacterial activity of lycosin-II, the MICs of lycosin-II against an *A. baumannii* strain were measured in the presence or absence of 5 mM of MgCl_2_, respectively. The growth–inhibitory curves were plotted by the method described above.

### 4.6. Hemolysis Assay

Fresh human red blood cells were obtained to measure the hemolytic activity of lycosin-II. Red cells were washed 3 times by PBS for 5 min at 3000 rpm. Cells were re-suspended by PBS at a final concentration of 1%. Then, peptides were mixed with equal volume cell suspension at final concentrations of 6.25–100 μM, while PBS and double-distilled water were added as negative and positive controls, respectively. After incubation for 1 h at 37 °C, mixtures were centrifuged for 5 min at 2000 rpm, and the absorbance of supernatant was measured at 570 nm by a microplate reader in 96-well microtiter plates. The percentage of hemolysis was calculated according to the following equation: % hemolysis = (A_sample_ − A_negative_)/(A_positive_ − A_negative_).

## Figures and Tables

**Table 1:** The MIC values of lycosin-II against clinical bacterial strains.

Bacterial strains	MIC (μM)
*Escherichia coli*	12.5
*Klebsiella pneumoniae*	50
*Streptococcus pyogenes *	50
*Staphylococcus saprophyticus*	3.1
*Staphylococcus epidermidis*	3.1
*Pseudomonas aeruginosa*	12.5
*Viridans Streptococci*	3.1
*Staphylococcus aureus* (three strains)	3.1
*Acinetobacter baumannii* (three strains)	3.1–6.3

## References

[1] Brogden K.A. (2005). Antimicrobial peptides: Pore formers or metabolic inhibitors in bacteria?. Nat. Rev. Microbiol..

[2] Narayana J.L., Chen J.Y. (2015). Antimicrobial peptides: Possible anti-infective agents. Peptides.

[3] Wang G., Li X., Wang Z. (2016). Apd3: The antimicrobial peptide database as a tool for research and education. Nucl. Acids Res..

[4] Wang G., Mishra B., Lau K., Lushnikova T., Golla R., Wang X. (2015). Antimicrobial peptides in 2014. Pharmaceuticals.

[5] Lee T.H., Hall K.N., Aguilar M.I. (2016). Antimicrobial peptide structure and mechanism of action: A focus on the role of membrane structure. Curr. Top. Med. Chem..

[6] Brown K.L., Hancock R.E. (2006). Cationic host defense (antimicrobial) peptides. Curr. Opin. Immunol..

[7] Hilchie A.L., Wuerth K., Hancock R.E. (2013). Immune modulation by multifaceted cationic host defense (antimicrobial) peptides. Nat. Chem. Biol..

[8] Sato H., Feix J.B. (2006). Peptide-membrane interactions and mechanisms of membrane destruction by amphipathic alpha-helical antimicrobial peptides. Biochim. Biophys. Acta.

[9] Shai Y. (2002). Mode of action of membrane active antimicrobial peptides. Biopolymers.

[10] King G.F., Hardy M.C. (2013). Spider-venom peptides: Structure, pharmacology, and potential for control of insect pests. Annu. Rev. Entomol..

[11] Pineda S.S., Undheim E.A., Rupasinghe D.B., Ikonomopoulou M.P., King G.F. (2014). Spider venomics: Implications for drug discovery. Future Med. Chem..

[12] Saez N.J., Senff S., Jensen J.E., Er S.Y., Herzig V., Rash L.D., King G.F. (2010). Spider-venom peptides as therapeutics. Toxins.

[13] Dubovskii P.V., Vassilevski A.A., Kozlov S.A., Feofanov A.V., Grishin E.V., Efremov R.G. (2015). Latarcins: Versatile spider venom peptides. Cell. Mol. Life Sci..

[14] Santos D.M., Verly R.M., Pilo-Veloso D., Maria M., de Carvalho M.A., de Cisalpino P.S., Soares B.M., Diniz C.G., Farias L.M., Moreira D.F. (2010). LyeTx I, a potent antimicrobial peptide from the venom of the spider lycosa erythrognatha. Amino Acids.

[15] Polyansky A.A., Vassilevski A.A., Volynsky P.E., Vorontsova O.V., Samsonova O.V., Egorova N.S., Krylov N.A., Feofanov A.V., Arseniev A.S., Grishin E.V. (2009). *N*-terminal amphipathic helix as a trigger of hemolytic activity in antimicrobial peptides: A case study in latarcins. FEBS Lett..

[16] Kuhn-Nentwig L., Sheynis T., Kolusheva S., Nentwig W., Jelinek R. (2013). *N*-terminal aromatic residues closely impact the cytolytic activity of cupiennin 1a, a major spider venom peptide. Toxicon.

[17] Pukala T.L., Doyle J.R., Llewellyn L.E., Kuhn-Nentwig L., Apponyi M.A., Separovic F., Bowie J.H. (2007). Cupiennin 1a, an antimicrobial peptide from the venom of the neotropical wandering spider cupiennius salei, also inhibits the formation of nitric oxide by neuronal nitric oxide synthase. FEBS J..

[18] Nomura K., Corzo G. (2006). The effect of binding of spider-derived antimicrobial peptides, oxyopinins, on lipid membranes. Biochim. Biophys. Acta.

[19] Kozlov S.A., Vassilevski A.A., Feofanov A.V., Surovoy A.Y., Karpunin D.V., Grishin E.V. (2006). Latarcins, antimicrobial and cytolytic peptides from the venom of the spider *Lachesana tarabaevi* (zodariidae) that exemplify biomolecular diversity. J. Biol. Chem..

[20] Yan L., Adams M.E. (1998). Lycotoxins, antimicrobial peptides from venom of the wolf spider lycosa carolinensis. J. Biol. Chem..

[21] Liu Z., Deng M., Xiang J., Ma H., Hu W., Zhao Y., Li D.W., Liang S. (2012). A novel spider peptide toxin suppresses tumor growth through dual signaling pathways. Curr. Mol. Med..

[22] Tan H., Ding X., Meng S., Liu C., Wang H., Xia L., Liu Z., Liang S. (2013). Antimicrobial potential of lycosin-I, a cationic and amphiphilic peptide from the venom of the spider *Lycosa singoriensis*. Curr. Mol. Med..

[23] Wang L., Wang Y.J., Liu Y.Y., Li H., Guo L.X., Liu Z.H., Shi X.L., Hu M. (2014). *In vitro* potential of lycosin-I as an alternative antimicrobial drug for treatment of multidrug-resistant *Acinetobacter baumannii* infections. Antimicrob. Agents Chemother..

[24] Liu Z.H., Qian W., Li J., Zhang Y., Liang S. (2009). Biochemical and pharmacological study of venom of the wolf spider *Lycosa singoriensis*. J. Venom. Anim Toxins.

[25] King J.D., Leprince J., Vaudry H., Coquet L., Jouenne T., Conlon J.M. (2008). Purification and characterization of antimicrobial peptides from the caribbean frog, leptodactylus validus (anura: Leptodactylidae). Peptides.

[26] Hancock R.E., Sahl H.G. (2006). Antimicrobial and host-defense peptides as new anti-infective therapeutic strategies. Nat. Biotechnol..

[27] Spellberg B., Bonomo R.A. (2013). “Airborne assault”: A new dimension in *Acinetobacter baumannii* transmission. Crit. Care Med..

[28] Lee H.Y., Chen C.L., Wu S.R., Huang C.W., Chiu C.H. (2014). Risk factors and outcome analysis of *Acinetobacter baumannii* complex bacteremia in critical patients. Crit. Care Med..

[29] Hagihara M., Housman S.T., Nicolau D.P., Kuti J.L. (2014). *In vitro* pharmacodynamics of polymyxin B and tigecycline alone and in combination against carbapenem-resistant *Acinetobacter baumannii*. Antimicrob. Agents Chemother..

